# Blood Coagulation Time in Disease

**Published:** 1908-01-04

**Authors:** 


### Points in Pathology.

### BLOOD COAGULATION-TIMES IN DISEASE.

We have referred to the simple microscope-slide method of determining the coagulability of the blood in a former issue. The method may be termed the Hinman-Sladen modification of Milian's slide method. It may be of interest to give a brief summary of the coagulation times that have been found, by this same method, in various diseases:—

*Catarrhal Jaundice.*—The cases of catarrhal jaundice investigated by Dr. Hinman and Dr. Sladen showed normal coagulation times, usually between five and a half and six minutes.

*Jaundice due to Gall Stones.*—The average time in a number of cases was eight minutes, so that the clotting power of the blood is not very abnormal in cholelithiasis. It will depend to some extent, of couse, upon the duration of the jaundice. The longest time observed was twelve and a half minutes.

*Jaundice due to Malignant Disease.*—In marked contrast to catarrhal and gall-stone jaundices comes malignant jaundice. This agrees with the well-known clinical fact that operations in cases of malignant jaundice are far more liable than are those for gall-stones to be followed by blood-oozing and severe and even fatal intestinal and other hæmorrhages. The average coagulation time in malignant jaundice was thirteen and a quarter minutes; the shortest time was nine minutes, and the longest twenty and a half minutes. The degree of delay in clotting will naturally vary not only with the depth and duration of the jaundice, but also with the nature and site of the neoplasm and its metastases, and with the condition of the patient.

*Urticaria.*—The coagulation time is frequently prolonged when the urticaria is acute, it being in many cases as long as nine and a half minutes.

*The Blood Diseases.*—It is rather surprising that in the blood diseases, in which spontaneous hæmorrhages or continuous oozing operations are so liable to occur, the coagulation times are often not abnormal. In the great majority of cases examined by Drs. Hinman and Sladen the time lay between four and a half and seven and a half minutes—that is to say, within the normal limits for the slide method.

In one case of pernicious anæmia it was as long as nine and a half minutes, and in another of acute lymphatic leukæmia without hæmorrhages it was eight and a quarter minutes. The majority of the cases of blood disease examined gave normal coagulation times, however. The tendency to hæmorrhage in these patients must therefore, it seems, have some other cause than deficient clotting power in the blood.

*Secondary Anæmia.*—Secondary anæmia is a somewhat loose term, applied by some only to those conditions in which the anæmia directly succeeds a blood loss, and by others to any anæmia which can be attributed to a definite cause whether that cause be blood loss or not. We ourselves mean by the term the anæmia which directly follows hæmorrhage. In two such cases, one of severe hæmaturia, the other of severe hæmorrhage in typhoid fever, the coagulation times were found to be very much increased—to fourteen and three-quarters and to fourteen minutes respectively.

*Aortic Aneurysm.*—Many aneurysm cases were tested, and not one was found in which the coagulation time was prolonged.

*Typhoid Fever.*—Prof. Wright and others have noted a delayed blood coagulation in the acute stages of typhoid fever, and an acceleration during convalescence. Some stress has been laid upon this as a possible factor in the tendency to hæmorrhage during the fever and to venous thrombosis later. The slide method, in the hands of Drs. Hinman and Sladen, gives some support to the facts noted by Prof. Wright. For example, the following are two series of observations in cases of typhoid fever:—

| | Clotting Time. 2nd to 3rd Week | Clotting Time. 3rd to 4th Week | Clotting Time. 5th to 6th Week |
|---|---|---|---|
| Case 1 | 12 mins. | $7\frac{1}{2}$ mins. | 3 mins. |
| Case 2 | $13\frac{1}{4}$ „ | $9\frac{1}{4}$ „ | $5\frac{1}{4}$ „ |

The opening of the vessels by the ulcerative processes in the bowel is, of course, the main factor in the hæmorrhages which occur, but the diminished coagulability of the blood doubtless delays the closure of the eroded vessels by blood clot. Increased coagulability of the blood during convalescence would not by itself cause thrombosis in the leg veins. Such thrombosis may occur without any increased coagulability at all, as in two cases where, concurrently with thrombosis, the times were six and a half and five and a half minutes. Nevertheless any increased coagulability of the blood must be an additional factor predisposing to thrombosis.

*Hæmophilia.*—Four cases of hæmophilia had coagulation times varying from eleven minutes to thirty-three minutes. The delay was constant and very marked. The effect of calcium in two of the cases was also great, the coagulation time coming down to six minutes, and the patients improving at once.

*Treatment by Calcium.*—It is well established that the administration of calcium by the mouth can increase the coaguability of the blood in cases in which the clotting is delayed abnormally. The two instances of hæmophilia just mentioned are examples of this. It has also been established that the giving of more than a certain amount of calcium has a reverse effect. It is further probable that calcium is not indicated when the blood-coagulation time is already normal.

It is important, therefore, first to determine the coagulation time and then to be guided by that as to whether calcium should be given or not. It does no harm if the dose is not excessive, and in a considerable number of cases it does demonstrable good. The iodide, the chloride and the lactate are all suitable forms. The lactate is as good as the other two; it is probably absorbed as chloride. The dose may be from $\frac{1}{2}$ to 4 drachms three times a day, and it is wise to interrupt its administration every few days, because its effects wear off if it is given continuously.

